# Digital radiography as an alternative method in the evaluation of bone density in uremic rats

**DOI:** 10.1590/2175-8239-JBN-2019-0008

**Published:** 2019-08-08

**Authors:** Bárbara Bruna Abreu de Castro, Wander Barros Carmo, Jasmin, Roberto Sotto Maior Fortes Oliveira, Vera Maria Peters, Vanda Jorgetti, Melani Ribeiro Custodio, Helady Sanders-Pinheiro

**Affiliations:** 1 Universidade Federal de Juiz de Fora, Laboratório de Nefrologia Experimental e Núcleo de Experimentação Animal, Juiz de Fora, MG, Brasil.; 2 Universidade Federal de Juiz de Fora, Divisão de Nefrologia e Núcleo Interdisciplinar de Estudos e Pesquisa em Nefrologia, Juiz de Fora, MG, Brasil.; 3 Universidade Federal de Juiz de Fora, Centro de Biologia da Reprodução, Juiz de Fora, MG, Brasil.; 4 Universidade de São Paulo, Faculdade de Medicina, Laboratório de Fisiopatologia Renal, São Paulo, SP, Brasil.

**Keywords:** Bone Density, Radiography, Disease Models, Animal, Renal Insufficiency, Chronic, Chronic Kidney Disease-Mineral and Bone Disorder, Densidade Óssea, Radiografia, Modelos Animais de Doenças, Insuficiência Renal Crônica, Distúrbio Mineral e Ósseo na Doença Renal Crônica

## Abstract

**Introduction:**

Digital radiography (DRx) may provide a suitable alternative to investigate mineral and bone disorder (MBD) and loss of bone density (BD) in rodent models of chronic kidney disease (CKD). The objective of this study was to use DRx to evaluate BD in CKD rats, and to evaluate the correlation between DRx findings and serum MBD markers and bone histomorphometry.

**Methods:**

Uremia was induced by feeding Wistar rats an adenine-enriched diet (0.75% for 4 weeks/0.10% for 3 weeks); outcomes were compared to a control group at experimental weeks 3, 4, and 7. The following biochemical markers were measured: creatinine clearance (CrC), phosphate (P), calcium (Ca), fractional excretion of P (FeP), alkaline phosphatase (ALP), fibroblast growth factor-23 (FGF-23), and parathyroid hormone (PTH). DRx imaging was performed and histomorphometry analysis was conducted using the left femur.

**Results:**

As expected, at week 7, uremic rats presented with reduced CrC and higher levels of P, FeP, and ALP compared to controls. DRx confirmed the lower BD in uremic animals (0.57±0.07 vs. 0.68 ± 0.06 a.u.; *p* = 0.016) compared to controls at the end of week 7, when MBD was more prominent. A severe form of high-turnover bone disease accompanied these biochemical changes. BD measured on DRx correlated to P (r=-0.81; *p* = 0.002), ALP (r = -0.69, *p* = 0.01), PTH (r = -0.83, *p* = 0.01), OS/BS (r = -0.70; *p* = 0.02), and ObS/BS (r = -0.70; *p* = 0.02).

**Conclusion:**

BD quantified by DRx was associated with the typical complications of MBD in CKD and showed to be viable in the evaluation of bone alterations in CKD.

## RESUMO

**Introdução:**

A radiografia digital (RxD) pode representar uma alternativa adequada para investigar o distúrbio mineral e ósseo (DMO) e a perda de densidade óssea (DO) em modelos de roedores da doença renal crônica (DRC). O objetivo deste estudo foi utilizar a RxD para avaliar a DO em ratos com DRC, e avaliar a correlação entre os achados da RxD e marcadores séricos de DMO e histomorfometria óssea.

**Métodos:**

A uremia foi induzida pela alimentação de ratos Wistar com dieta enriquecida com adenina (0,75% por 4 semanas/0,10% por 3 semanas); os resultados foram comparados com um grupo controle nas semanas experimentais 3, 4 e 7. Os seguintes marcadores bioquímicos foram medidos: clearance de creatinina (CCr), fosfato (P), cálcio (Ca), fração excretada de P (FeP), fosfatase alcalina (ALP), fator de crescimento de fibroblastos-23 (FGF-23) e paratormônio (PTH). A imagem da RxD foi obtida e a análise histomorfométrica foi realizada com o fêmur esquerdo.

**Resultados:**

como esperado, na semana 7, os ratos urêmicos apresentaram redução do CCr e níveis mais altos de P, FeP e ALP em comparação aos controles. A RxD confirmou a menor DO em animais urêmicos (0,57 ± 0,07 vs. 0,68 ± 0,06 u.a.; *p* = 0,016) em comparação aos controles no final da semana 7, quando a DMO foi mais proeminente. Uma forma grave de doença óssea de alta renovação celular acompanhou essas mudanças bioquímicas. A DO, medida na RxD foi correlacionada a P (r = -0,81; *p* = 0,002), ALP (r = -0,69, *p* = 0,01), PTH (r = -0,83, *p* = 0,01), OS/BS (r = -0,70 *p* = 0,02) e Ob.S/BS (r = -0,70; *p* = 0,02).

**Conclusão:**

A DO quantificada por RxD esteve associada às complicações típicas da DMO na DRC e mostrou-se viável na avaliação de alterações ósseas na DRC.

## INTRODUCTION

In humans, trabecular bone has a turnover rate approximately 8 times faster than that of compact bone, and it is highly responsive to metabolic stimuli^1^. This high turnover rate makes trabecular bone the primary site for the detection of early bone loss that precedes fractures, and for monitoring the efficacy of different treatments aiming to prevent or slow down bone loss in clinical settings^2^. Thus, the evaluation of bone density (BD) is considered a major clinical tool for the detection of pathologies affecting bone structure^3^.

Bone density is assessed using densitometry methods that are based on the principle of differential absorption of photons by tissues of different radiodensities, as well as by different regions of varying radiodensities within the same tissue. The absorption of photons is directly related to the thickness and composition of the bone tissue. Therefore, as BD decreases, less photons are absorbed, resulting in the attenuation of the radiographic signal^4^^,^ 5.

Dual-energy X-ray absorptiometry (DXA) is the most used densitometry method to evaluate bone quantitatively in the general population, as it is non-invasive, uses low doses of radiation, and provides BD with high precision and good sensitivity and specificity. Quantitative computed tomography (QCT) is being increasingly used as an alternative method, sharing the same attributes as DXA but with the added advantage of providing separate assessment of cortical and trabecular BD^4^^-^ 6.

The trabecular bone score is a new method that estimates the trabecular microarchitecture from DXA images. Studies in humans confirm its association with the findings of QCT and with trabecular bone assessment by bone histomorphometry, making this a promising method for the evaluation of fracture risk in individuals with and without renal disease^7^^,^ 8. In patients with chronic kidney disease (CKD), histomorphometry is considered the gold standard for the assessment of BD^4^^,^ 6. As histomorphometry is highly invasive, alternative non-invasive methods are being studied, combining imaging techniques with analysis of bone turnover biomarkers in order to assess fracture risk in patients with CKD^5^^,^ 9.

In experimental studies, the digital radiography (DRx) has also been used for the evaluation of BD^10^^,^ 11. With DRx, digital images are obtained electronically, converted to numerical data using a custom software, sampled, and stored for off-line analysis. For analysis, the digital image is divided into pixels, and the grey scale tone for each pixel is numerically coded. Thus, each pixel is associated with a number representing the color of in the area or the intensity of gray tones. In this way, the image is converted into a set of numbers and can be visualized on a computer screen or be printed for analysis^12^^,^ 13. To date, DRx has not been used for analysis of mineral and bone disorder (MBD) in experimental models of CKD.

The objective of this study was to evaluate BD in uremic rats using DRx, and to evaluate the association of image-based measures with the biochemical markers of renal MBD and with aspects of bone histomorphometry.

## METHODS

### STATEMENT OF ETHICS

All procedures were performed in accordance with the Brazilian Federal Law, (11,794, October 8^th^ 2008) and the guidelines of the National Council for the Control of Animal Testing. The study was approved by the Ethics Committee of Animal Use of Federal University of Juiz de Fora, Protocol number 031/2013.

### EXPERIMENTAL PROTOCOL AND DIETS

**Figure 1:**
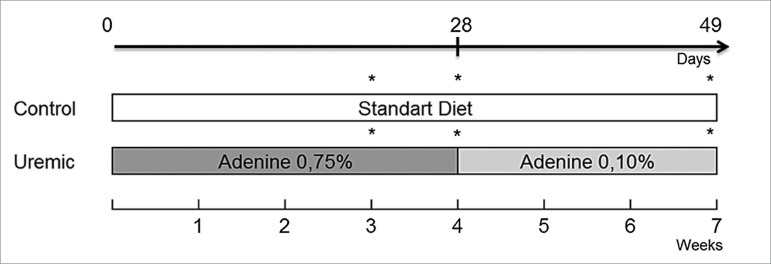
From day 0 to day 49 (7 weeks), standard diet was administered to the Control group. From day 0 to day 28 (4 weeks), 0.75% adenine diet was administered to the Uremic group. At the day 28, the 0.75% adenine diet was stopped, and from day 28 to day 49 (i.e., 3 weeks), a 0.1% adenine diet was administered to the Uremic group. Body weight, food and water intake were measured once a week. Stars indicate the time-points of measurement of water intake, urine volume, and euthanasia (N = 8).

Male Wistar rats 8 to 12 weeks old, weighing 200g to 300g were used. The animals were obtained from the Biology Center of the Federal University of Juiz de Fora and randomly divided into two groups (Control and uremic groups,) containing 24 animals each. The animals in the Control group were fed a standard diet (Pragsoluções, Jau, Brazil) until the end-point of 7 weeks. Uremic group animals were fed a 0.75% adenine enriched-diet for 4 weeks followed by a 0.10% adenine enriched-diet in the following 3 weeks (Pragsoluções) (Figure 1).

Eight animals from each group were euthanized at experimental weeks 3, 4, and 7. The day before euthanasia, animals were housed in metabolic cages for a 24-hr urine collection. The animals were anesthetized with xylazine (10 mg/kg) and ketamine (90 mg/kg) (König, Avellaneda, Argentina), IP. Blood samples were collected by cardiac puncture. Left femurs were removed for DRx analysis and bone histomorphometry.

### BIOCHEMICAL PARAMETERS

At weeks 3, 4 and 7, serum creatinine, P, ALP, and Ca, and urinary creatinine and P levels were determined using an automatic analyzer, LabMax Progress (Labtest Diagnostica SA, Lagoa Santa, Brazil). Additionally, serum parathyroid hormone (PTH) (Rat Intact PTH ELISA kit, Immutopics, San Clemente, USA) and fibroblast growth factor-23 (FGF-23) (FGF-23 ELIZA kit, Clone Cloud Corp. Houston, USA) assays were performed by ELISA (R & D Systems, Minneapolis, USA).

### BONE HISTOMORPHOMETRY

The left femur of 5 animals per group was removed, dissected free of soft tissue, immersed in 70% ethanol, and processed as described previously^14^. Static, structural, and dynamic parameters of bone formation and resorption were measured in distal metaphysis (magnification 250x; 30 fields), 195 µm from the epiphyseal growth plate, using an OsteoMeasure image analyzer (Osteometrics, Atlanta, GA, USA). Structural parameters included trabecular thickness (in **µm**), trabecular separation (in **µm**), and trabecular number (in trabeculae/mm). The indices of static formation included the proportion of trabecular bone volume and osteoid volume to total bone volume (both in %), osteoid thickness (in **µm**), and osteoid/osteoblast surfaces (both in % of bone surface). The indices of static resorption included eroded surface and osteoclast surface (both in % of bone surface). Mineral apposition rate was determined from the distance between the two tetracycline labels, divided by the time interval between the two tetracycline administrations and expressed in µm/day. Mineralization lag time was expressed in days. The percentage of double tetracycline-labeled (mineralizing) surface per bone surface and bone formation rate completed the dynamic evaluation. Results are also described according to the turnover mineralization volume (TMV) classification^15^. Histomorphometric indices were reported using the nomenclature recommended by the American Society of Bone and Mineral Research^16^. All animal data were obtained by examiners blinded to the study protocol.

### EVALUATION OF BD BY DRX

For DRx analysis (direct method), the left femur of 6 animals per group was fixed in 100% ethanol. Images of the whole bone were then captured using a Kodak In vivo Image Station PRO, equipped with a CCD camera (Carestream Health Inc., Rochester, NY). The DRx images were obtained using the following parameters: 60 s exposure time, 2x2 binning, KVP35, 0.8 mm aluminum filter, 2.8 f-stop, and 80 mm field of vision. The acquired images were analyzed using the Carestream MI Application software (version 5.0.2.30, Carestream Health Inc.). After image calibration to optical density, the bone area was delimited using the automatic selection tool and the mean grey intensity for each pixel in the delimited area quantified in arbitrary units (a.u.)^11^^,^ 13 ^,^ 17.

### STATISTICAL ANALYSIS

The data are reported as mean ± standard deviation or median with minimum and maximum, as appropriate for the data distribution evaluated using the Kolmogorov-Smirnov test of normality. Comparisons between biochemical parameters of CKD MBD for the Uremic and Control groups were performed using Student’s *t*-test. Pearson’s and Spearman’s correlation coefficients were used to evaluate the relationship between BD, measured by DRx, and biochemical parameters and aspects of bone histomorphometry at week 7. Statistical significance was set at a *p*-value < 0.05, and all analyses were performed using SPSS statistical software (Version 21; IBM Corporation, Chicago, IL).

## RESULTS

### MORTALITY AND BIOCHEMICAL PARAMETERS

**Figure 2:**
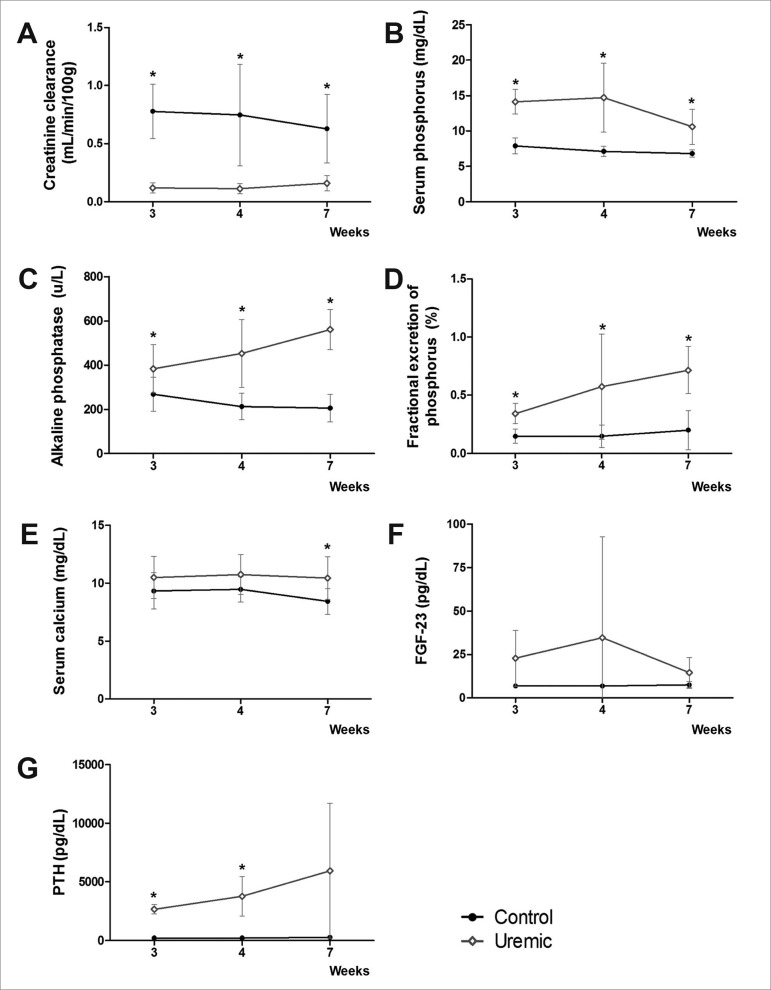
Biochemical parameters collected at weeks 3, 4 and 7: (A) Creatinine clearance (CrC), (mL/min/100g), (B) Serum phosphate (P) (mg/dL), (C) Alkaline phosphatase (ALP) (u/L), (D) Fractional excretion of P (FeP) (%), (E) Serum Calcium (Ca) (mg/dL), (F) fibroblast growth factor-23 (FGF-23) (pg/dL) and (G) Parathyroid hormone (PTH) (pg/dL). Data are expressed as mean ± SD for each group, and Student’s *t*-test used to compare data at each week, *p < 0.05.

One animal died in the Uremic group at week 3. The results of the adenine-enriched diet on CrC are shown in Figure 2A. CrC was markedly decreased in the Uremic group at week 4, following the initial diet with 0.75% adenine enrichment for 4 weeks, with levels of 0.11 ± 0.04 mL/min/100g compared to 0.75 ± 0.44 mL/min/100g for animals in the Control group (*p* = 0.016). This renal dysfunction in the Uremic group persisted for 3 weeks after the reduction of dietary adenine concentration to 0.1%, with CrC levels at week 7 of 0.16 ± 0.06 mL/min/100g compared to 0.62 ± 0.29 mL/min/100g for the Control group (*p* = 0.01).

Renal dysfunction was associated with MBD characterized by specific biochemical abnormalities. Hyperphosphatemia was found in the Uremic animals at the 3 time-points (Figure 2B). P levels in the Uremic group doubled compared to animals in the Control group at week 4, with levels of 14.70 ± 4.87 mg/dL and 7.11 ± 0.71 mg/dL, respectively (*p* = 0.01). P levels remained high at week 7, with values of 10.61 ± 2.49 mg/dL and 6.82±0.53 mg/dL for the Uremic and Control groups, respectively (*p* = 0.001) (Figure 2B).

ALP was statistically higher in the Uremic group compared to the Control group, at all 3 time-points (Figure 2C). In the Uremic group, ALP gradually increased to 383.14 ± 109.55 u/L at week 3, 452.71 ± 153.43 u/L at week 4, and 561 ± 90.90 u/L at the end-point of the experiment (week 7). The fractional excretion of P (FeP) increased while the renal function worsened. The FeP levels were elevated for animals in the Uremic group compared to the Control group, at the 3 time-points (Figure 2D). FeP levels in Uremic animals increased to 34.21 ± 8.63% and 71.59 ± 20.23% at weeks 3 and 7, respectively, compared to 20.06 ± 16.83% at week 7 for animals in the Control group. While Ca levels were comparable between groups at weeks 3 and 4, levels were elevated for animals in the Uremic group at week 7 (Figure 2E).

An increase in FGF-23 levels was expected to parallel the increase in renal dysfunction. In our animal model, however, levels of FGF-23 presented a tendency for higher value only at week 3 (Figure 2F). While PTH levels were widely variable in the Uremic group, levels were consistently higher compared to the Control group at weeks 3 and 4 (Figure 2G). Comparative values were of 2,659.20 ± 392.57 pg/dL vs. 214.84 ± 53.31 pg/dL (*p* = 0.001), respectively, for the Uremic and Control groups at week 3, and 3,769.40 ± 1693.62 pg/dL vs. 214.84 ± 53.31 pg/dL (*p* = 0.01), respectively, at week 4. At week 7 there was only a tendency for PTH levels to be higher in the Uremic group (5,940.2 ± 5,740.35 pg/dL vs. 272.3 ± 192. 22 pg/dL; *p* = 0.09) (Figure 2G).

### BONE HISTOMORPHOMETRY

Bone histomorphometry revealed that structural bone parameters did not differ between groups (Table 1). The bone formation rate (BFR/BS) was significantly higher in the CKD group than in the Control group. Other parameters of bone formation, such as osteoid (OS/BS), osteoclast (Oc.S/BS), and osteoblast (Ob.S/BS) surfaces were also significantly higher in the CKD group (Table 1). These findings confirmed the achievement of a high-turnover bone disease.

**Table 1:** Bone histomorphometry data after 7 weeks^a^

Bone parameters	Control	Uremic	*p*
*Structural parameter*			
Trabecular Volume (BV/TV, %)	22.64 ± 3.71	25.69 ± 15.83	0.686
Trabecular Number (Tb.N, mm)	4.20 ± 0.35	4.46 ± 2.48	0.810
Trabecular Thickness (Tb.Th, µm)	54.03 ± 8.57	57.78 ± 17.92	0.680
Trabecular Separation (Tb.Sp, µm)	185.25 ± 18.63	265.23 ± 229.92	0.434
*Formation parameter*			
Osteoid Thickness (O.Th, µm)	0.97 (0.49 - 1.46)	4.99 (2.63 - 25.68)	0.077
Osteoid Surface (OS/BS, %)	1.87 ± 1.13	33.20 ± 21.33	0.010
Osteoblast Surface (Ob.S/BS, %)	1.71 ± 0.97	22.79 ± 11.67	0.007
Mineralizing Surface (MS/BS, %)	1.58 ± 0.77	2.59 ± 0.86	0.139
Mineral Apposition Rate (MAR, µm/day)	0.37 ± 0.20	1.32 ± 0.66	0.021
Bone Formation Rate (BFR/BS, µm3/µm2/day)	0.01 (0.0 - 0.01)	0.03 (0.02 - 0.04)	0.004
*Resorption parameter*			
Eroded Surface (ES/BS, %)	3.67 ± 0.91	14.20 ± 7.42	0.017

a Data expressed as mean ± SD and Student’s t-test used to compare groups or as median (min-max) and Mann-Whitney test used to compare groups (N=5).

### EVALUATION OF BD BY DRX

**Figure 3:**
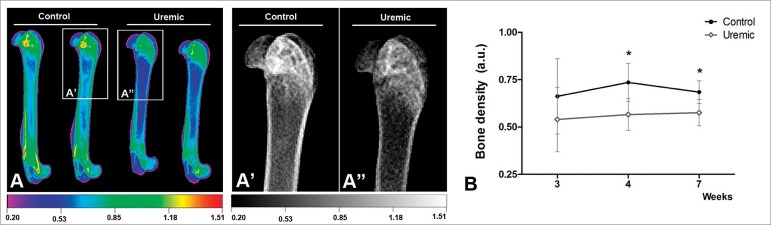
(A) Representative Digital Radiography (DRx) of left femurs from each group, presented in colour scale. (A’) Representative DRx in grey tones for the femur of an animal in the Control group, and (A’’) Representative DRx grey tones for the femur of an animal from the Uremic group. (B) Bone density calculated from the mean intensity of grey tones (a.u.). Data are expressed as mean ± SD in each group and Student’s *t*-test used to compare data at each week, *p < 0.05.

Comparative qualitative analysis of BD images of the left femurs of animals from the Uremic and Control groups are shown in Figure 3A e 3B. The left femur of Uremic animals showed several areas of severe bone reabsorption, indicative of bone rarefaction. Areas of diminished cortical bone thickness and expansion of trabecular bone were identified surrounding regions of rarefaction (Figure 3A”). Optical density analysis of the entire femur (Figure 3B) indicated significantly lower BD in the Uremic group, with BD values of 0.57 ± 0.08 a.u. compared to 0.73 ± 0.10 a.u. for the Control group (*p* = 0.01) at week 4, and 0.58 ± 0.07 a.u. vs 0.68 ± 0.06 a.u., respectively, at week 7 (*p* = 0.016).

### ASSOCIATION BETWEEN BD AND ASPECTS OF BONE HISTOMORPHOMETRY AND BIOCHEMICAL PARAMETERS AT WEEK 7

**Figure 4:**
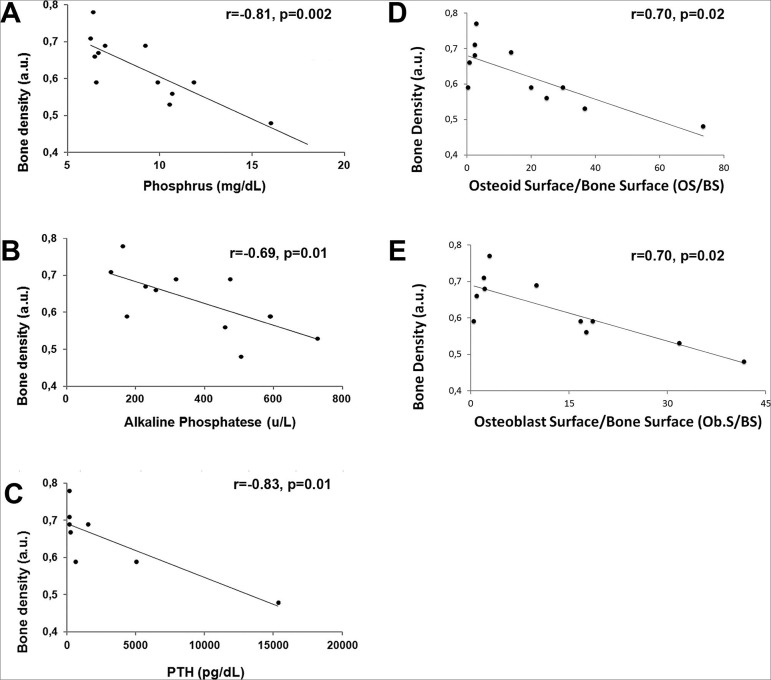
Pearson´s and Spearman’s correlation between bone density measured by Digital Radiography (DRx) and biochemical parameters at week 7: (A) Correlation between bone densisty (BD) and Serum phosphate (P); (B) Correlation between BD and alkaline phosphatase (ALP); and (C) Correlation between BD and Parathyroid hormone (PTH); (D) Correlation between BD and Osteoid Surface/Bone Surface (OS/BS); (E) Correlation between BD and Osteoblast Surface/Bone Surface (Ob.S/BS).

Results of Pearson’s and Spearman’s correlation analysis, evaluating the association between BD, measured by DRx, and selected aspects of bone histomorphometry and selected biochemical parameters of MBD in CKD are shown in Figure 4. The analysis revealed a significant inverse correlation between BD and P (r = -0.81; *p* = 0.002) (Figure 4A), ALP (r= -0.69, *p* = 0.01) (Figure 4B), PTH (r = -0.83, *p* = 0.01) (Figure 4C), OS/BS (r = -0.70; *p* = 0.02) (Figure 4D), and Ob.S/BS (r = -0.70; *p* = 0.02) (Figure 4E).

## DISCUSSION

CKD induces significant change in bone remodeling mechanisms, resulting in an imbalance between bone formation and reabsorption. The resulting changes in mineral organic content of bone can be measured by various methods^5^^,^ 18. To our knowledge, the application of DRx for analysis of BD in uremic experimental models has not been previously evaluated. Recently, the analysis of BD in patients with CKD has gained importance in clinical practice because of its association with increased risk for fractures, and increased overall and cardiovascular mortality^19^^,^ 20.

We adapted the adenine model as previously described^21^. Damment et al. modified the original protocol of feeding animals with 0.75% adenine for 4 weeks, by adding a maintenance phase consisting of a 0.1% adenine diet for 18 weeks^21^. In our study, we used the initial 4 weeks of 0.75% adenine feeding and added 3 weeks of 0.1% adenine feeding. Our adapted adenine feeding protocol aimed to extend the period of renal dysfunction while reducing mortality^21^.

In our experimental model, glomerular filtration rate (GFR), estimated by CrC, was reduced by 75% from normal values in the Uremic group at the end of the experiment. Extrapolating our data to The Kidney Disease: Global Improving Outcomes (KDIGO) classification of CKD, we can infer that the GFR reduction in Uremic animals was equivalent to patients with category 4^22^.

MBD is common at this category of experimental CKD^23^. However, CKD MBD sings are present early in the course of CKD, with abnormal serum levels of P, Ca, FGF-23, PTH, and calcitriol, which precede identifiable changes in BD^24^. These early changes were demonstrated by Pereira et al., who reported increased FGF-23 production by osteocytes in patients with category 2 CKD^25^. In the transition from category 2 to 3, the reduction of GFR promotes a P overload, which stimulates the secretion of FGF-23 and increases phosphaturia^26^^,^ 27. As CKD progresses, increased levels of FGF-23 reduce calcitriol levels and, consequently, increase levels of PTH in an attempt to maintain P homeostasis^24^^,^ 28. The biochemical changes, mainly hyperphosphatemia, are most evident when patients progress to category 4 CKD^29^.

In addition to the reduction in CrC, our data reproduced the kinetics of both early and late biochemical indicators of CKD MBD; early biochemical markers included a rise in FeP and FGF-23 levels, with elevation of PTH levels and hyperphosphatemia as late markers. In experimental models, these biochemical changes, and hyperphosphatemia more specifically, have been correlated to an increased prevalence of vascular calcification, bone disorders, and mortality^30^^-^ 32. ALP is another biochemical marker of CKD that has traditionally been linked to bone remodeling and cardiovascular risk in uremic patients, also augmented as the renal function decreases^33^^,^ 34.

We believe that the absence of significant elevation of FGF 23 in our study was due to the large intra-group variation of uremic animals in the fourth week. In addition, we observed that serum calcium levels increased during CKD onset. A potential explanation could be the peculiar feature of the experimental model. Adenine can induce elevation of serum calcium, as described in other studies, because in addition to inducing CKD, it acts directly on osteoblasts compromising their mineralization capacity, inducing a severe bone disease with high remodeling rate^35^^-^ 37.

Bone abnormalities in animal models have predominantly been associated to elevated levels of PTH^36^^,^ 38. In our model, PTH levels increased during the experiment, but levels were statistically different to the Control group only at the early time-points of measurement. The absence of between-group differences at later time-points likely reflects the large between-animal variability in PTH levels in CKD^36^.

Patients with CKD have a high prevalence of fractures when compared to the general population, which shows that renal dysfunction increases the risk for this condition^19^^,^ 20. After the 2012 KDIGO guidelines, the evaluation of BD has increasingly been included as a component of the clinical investigation of CKD patients, justified by the importance of early diagnosis of fracture risk and the need to evaluate new therapies aiming to preserve bone mass in this population^9^.

The risk of bone fracture is defined as a bone’s capacity to maintain its structure under an applied force. This capacity is directly related to BD, bone quality, and bone remodeling rate^39^. BD is the fraction of bone than can be quantified. While commonly evaluated by DXA, BD has more recently been evaluated by QCT, which discriminates cortical and trabecular bone^5^^,^ 40. However, rapid and less expensive techniques, like DRx, can also be applied to assess trabecular bone mass and the more general features of disease-related changes in BD. DRx has the advantage of being easier to use than DXA and QCT and more feasible for basic research^13^, beeing increasingly used to provide surrogate measures of BD in small animals^10^^,^ 11 ^,^ 13 ^,^ 41 ^,^ 42. DRx imaging was reported to be comparable to other imaging techniques (DXA and QCT), biochemical markers, and histological patterns for differentiating normal and developing bone^10^^,^ 13, as well as for identifying bone effects of various conditions including diabetes mellitus^42^, titanium implants^41^, disuse-induced bone loss^11^, and osteoporosis^43^. Studies on whole bone assessment of MBD in experimental models of CKD were not found in the literature. In our experiments, lower BD measured by DRx was found in uremic animals at all measurement time-points, and BD changes correlated to changes in biochemical markers of MBD featured by the elevation of PTH, P, and ALP and to changes of bone histomorphometry characteristic of a high-turnover bone disease. Therefore, there was a direct link between reduced BD and high-turnover bone disease found in CKD^44^. The absence of correlation between the trabecular volume and BD can be explained by the unique features of the adenine model. Adenine acts directly on osteoblasts compromising the cells capacity of mineralization and inducing a relative hypercalcemia and a high-turnover bone disease^35^^-^ 37. In our study, we found an increased osteoid surface evidencing compromised bone calcification; however, we also identified high bone reabsorption characterized by the increased osteoclast surface. Therefore, in the absence of bone tissue mineralization, BD measured by Rx is lower than that found in control animals.

The limitations of our study should be acknowledged. While previous studies in rodents have demonstrated a good correlation of DRx findings with DXA and QCT results, as well as with serum markers of bone disease, inclusion of another imaging technique for comparison would have strengthened the findings of our study^10^.

## CONCLUSION

In conclusion, DRx was able to detect BD reduction in femurs of uremic rats and results were associated with markers of CKD-related high-turnover bone disease. Thus, DRx is a helpful tool in the study of BD in animal models of CKD.
